# Investigation of the differential susceptibility of extraocular muscles in patients diagnosed with ocular myasthenia gravis based on the computerized diplopia test and the Ocular Motor Nerve Palsy Scale

**DOI:** 10.3389/fneur.2024.1353248

**Published:** 2024-05-30

**Authors:** Yi-fei Fan, Sai-jun Tu, Yani Liu, Xue-mei Li, Tie-juan Liu, Ling-yun Zhou

**Affiliations:** ^1^Ocular Motility Disorder Treatment & Rehabilitation Center, Department of Acupuncture, Harbin Medical University, Harbin, Heilongjiang, China; ^2^The First Affiliated Hospital of Harbin Medical University, Harbin, Heilongjiang, China

**Keywords:** ocular myasthenia gravis, extraocular muscle, diplopia, ptosis, computerized diplopia test

## Abstract

**Introduction:**

The pattern of extraocular muscle involvement in ocular myasthenia gravis varies across different reports, diverging from our own observations. Thus, we employed two novel tools to discern this pattern.

**Methods:**

A retrospective analysis was conducted to collect and organize clinical data from 43 patients diagnosed with ocular myasthenia gravis. Each patient underwent both the computerized diplopia test and the Ocular Motor Nerve Palsy Scale assessment to evaluate the involvement of extraocular muscles.

**Results:**

Among the patients, there were 30 male and 13 female individuals, with a total of 113 affected extraocular muscles identified. Among all the affected extraocular muscles, the involvement of the levator palpebrae superioris muscle accounted for 35.40%, medial rectus muscle 7.7%, lateral rectus muscle 16.81%, superior rectus muscle 13.27%, inferior rectus muscle 12.39%, superior oblique muscle 1.77%, and inferior oblique muscle 2.65% of the total affected extraocular muscles. The positivity rates of the Neostigmine test were 89.19%, AChR antibody detection was 59.38%, and repetitive nerve stimulation was 34.38%. The AChR antibody positive rate among patients with only diplopia was 100%; among those with only ptosis, it was 80%; and among those with both diplopia and ptosis, it was 86.67%.

**Conclusion:**

The involvement of the extraocular muscles is not uniform. The levator palpebrae superioris exhibits the highest incidence rate, followed by the four rectus muscles and two oblique muscles. The inferior oblique involvement typically occurs when four or more EOMs are affected. Moreover, the levator palpebrae superioris and medial rectus show a higher tendency for bilateral involvement compared with other extraocular muscles.

## 1 Introduction

Myasthenia gravis (MG) is an autoimmune disease characterized by the involvement of postsynaptic acetylcholine receptors at the neuromuscular junction, resulting in muscle weakness or fatigue that can manifest as either localized or generalized symptoms (1). The global incidence of MG is approximately 1.7 million individuals per year (2). The Myasthenia Gravis Foundation of America (MGFA) (1) defined ocular myasthenia gravis (OMG) as a subtype characterized by weakness of the extraocular muscle (EOM) while maintaining normal strength in other muscle groups. Symptoms of OMG include unilateral or bilateral ptosis with or without impairment of ocular motility or diplopia. Among patients with MG, approximately 75% initially present with diplopia and 60% exhibit symptoms specific to EOMs, with 80% of them progressing to generalized myasthenia gravis (GMG) (3–6).

The higher susceptibility of EOMs in OMG has been explored by various studies, offering explanation from different perspectives. Several factors have been considered to contribute to this differential susceptibility. These factors include the unique immune environment in which EOMs are located (7), the exceptional precision required by EOMs for human vision, the high-frequency discharge of oculomotor neurons (8), distinctive complement regulation compared with other muscle groups (9, 10), variations in acetylcholine receptor (AChR) subtypes in EOMs (11), the smaller size of the postsynaptic membrane (12), and differences in muscle fiber types between EOMs and skeletal muscles (10). While these factors have provided valuable insights, it is important to note that they cannot fully elucidate the varying susceptibility of different EOMs in OMG. Further research is needed to investigate additional mechanisms and factors that may contribute to the observed differences. Understanding the specific susceptibility patterns of EOMs in OMG is crucial for enhancing our knowledge of the disease and developing more targeted and effective diagnostic and treatment approaches.

Currently, research studies on OMG have identified differences in the involvement of EOMs, but there is still no definitive conclusion regarding the specific susceptibility of each EOM. Marie Cleary (13) pointed out that bilateral and symmetrical involvement, mainly affecting the superior rectus (SR), could be the pattern of EOM involvement in her study. Oosterhuis (14) found that the medial rectus (MR) is often the first to be involved. Vertical gaze, particularly elevation, is also said to be frequently affected. Junjie Tang (15) concluded that horizontal diplopia was the initial symptom in most adult OMG patients, and the lateral rectus (LR) was the most susceptible muscle. However, their conclusions significantly deviate from our clinical observations. We recognize that this disparity may be attributed to differences in the method they employed for assessing EOM involvement compared to our approach.

This study innovatively utilizes a computerized diplopia test (CDT) as the primary tool for detecting diplopia and uses the Ocular Motor Nerve Palsy Scale (OMNPS) to quantify the degree of eyelid ptosis and the range of eye movements. CDT (16), which is based on the principle of the Hess screen test, combines the formation and interpretation theory of the red glass test and Lancaster screen test, with the help of computer technology; CDT is not only as accurate as the Hess test but is also more practical for clinical use. CDT utilizes the maximum of diplopia from a condensed plot to assess the severity of diplopia based on the functionality of yoke muscles. This approach effectively circumvents limitations related to screen size and is comparably precise to a diffuse plot. The CDT test automatically generates results containing the amplitude of deviations and displacement between the standard point and subjective recorded position across nine gaze directions. The integration of image and data results facilitates clinicians in making more intuitive and precise assessments of patients’ affected EOMs.

**Figure 1:**
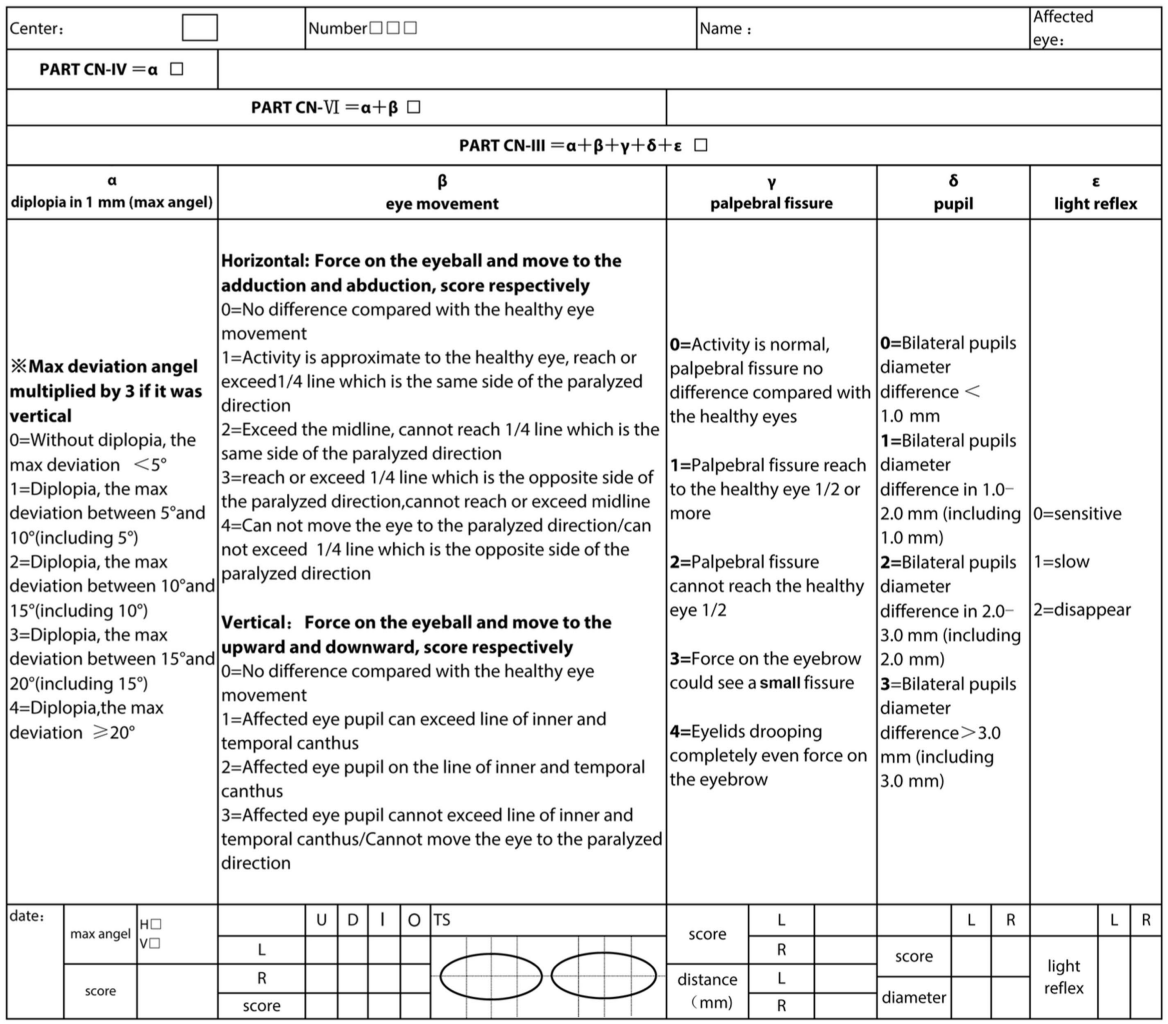
Details of the Ocular Motor Nerve Palsy Scale. L, Left; R, right; TS, total score; U, upside; D, downside; I, inner side; O, outside; H, horizontal; V, vertical.

OMNPS (17, 18) is a scale that contains five main items, diplopia at 1 m, eye movement condition, palpebral fissure, pupil size, and light reflex (Figure 1). The OMNPS shows high content validity, construct validity, inter-rater reliability, test–retest reliability, and internal consistency. It is therefore suitable to be used as a clinical scale to evaluate the severity of ocular motor nerve palsy. Combining both examination methods can maximize the objectivity and accuracy of the results. Before the introduction of OMNPS, methods for quantifying the severity of extraocular muscle paralysis were relatively crude, such as the QMGS extraocular muscle section, Maddox rod, and prism diopter, which suffered from shortcomings such as inadequate quantification and incomplete metrics. OMNPS, however, accurately records precise data including the range of eyeball movement in horizontal and vertical directions, pupil size, and distance of eyelid drooping.

## 2 Methods

This study employed a retrospective case series design. We reviewed the records of consecutive patients with OMG identified from a diagnostic index referred to one consultant neurologist (LY Z), with expertise in neuro-ophthalmology, at the Institute of Department of Oculomotor Disorders in the First Affiliated Hospital of Harbin Medical University between January 2020 and January 2022. The diagnosis of OMG was made based on ① the presence of symptoms such as ocular muscle weakness (ptosis and/or diplopia) that alleviated after rest and worsened after activity; ② at least one positive result from the following tests was required: Neostigmine test, repetitive electrical stimulation, or serum acetylcholine receptor antibody. Although not all patients underwent all investigations, all medical records should contain the results of CDT and OMNPS. All patients signed their informed consent, and the study was approved by the Ethics Committee of the First Affiliated Hospital of Harbin Medical University of China (approval number: 201452).

**Figure 2:**
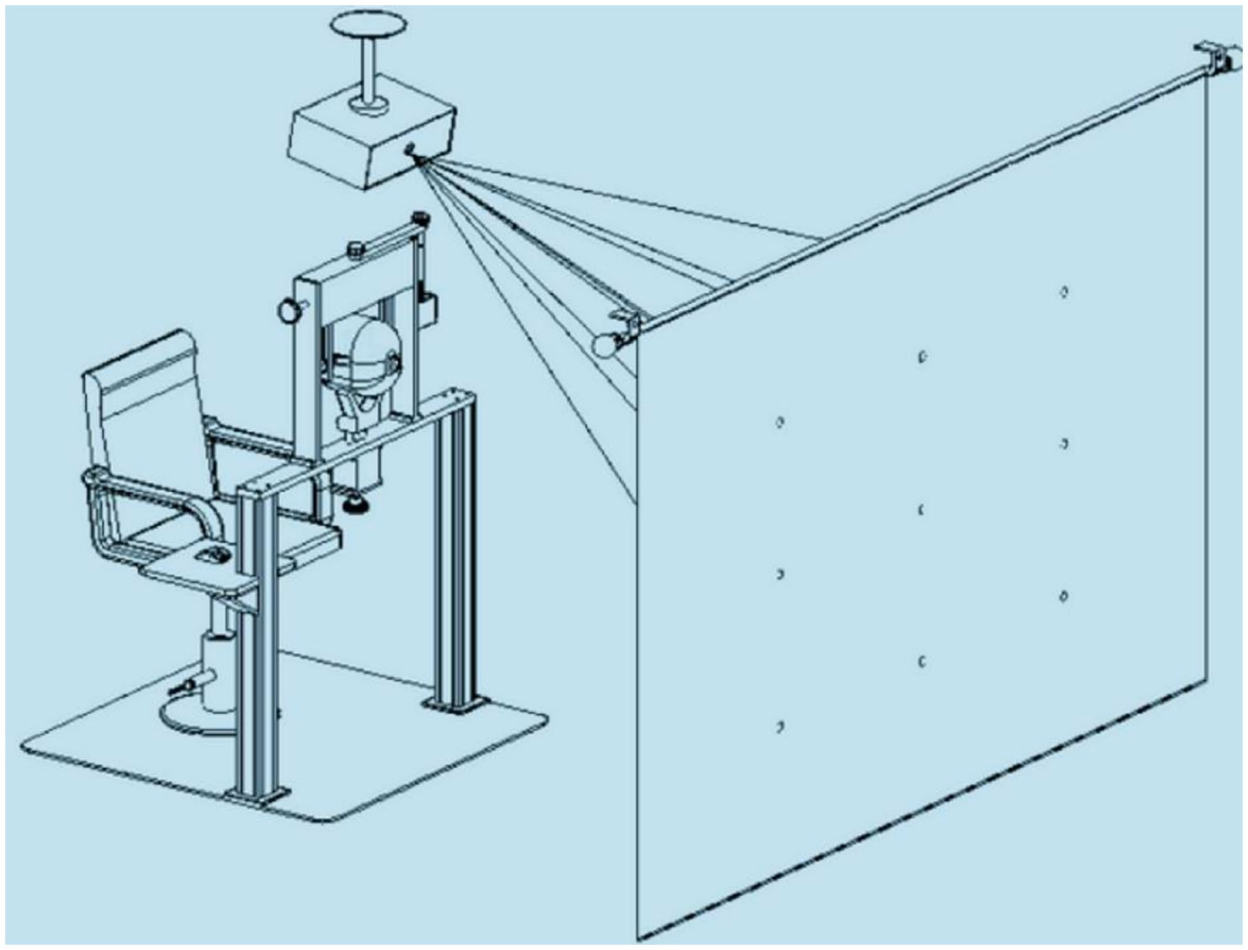
Schematic diagram of the device for applying the computerized diplopia test.

CDT (16): The device for applying the test contained three parts, namely, a head fixation frame, a computer with a projection system, and software for the test (Figure 2). Interactive software was based on Windows, while the test mode was as follows: 1 m working distance and 20° of ocular rotation from each target to the primary position. During the test, the patient wore red-blue glasses, and then a red wafer appeared on 1 of the 9 gaze positions. The patient was asked to align the red wafer with the blue circle cursor by moving the mouse. When the patient believed that they had aligned the red wafer with the blue circle, they clicked the left mouse button, and then the next point appeared. After testing all nine positions, the program switched the color of the wafer and circle and repeated the same test procedure. All participants who enrolled to determine the interobserver agreement were guided by the same doctor and self-paced to complete the test. The test results were stored in the plot form, and the amplitude of deviations and displacement between the standard point and subjective recorded position in nine gaze directions were generated automatically. The inspection results will be jointly interpreted by Y W and XM L, and disputed ones will be handed over to LY Z for final determination.

OMNPS (18): After head fixation, patients were asked to move the eyeball toward the left and right and straight up and down. The functional eye movement in four directions was observed and graded according to the scoring criteria. The frontal muscle above the eyebrow was fixed and did not affect the activity of the upper eyelid. Patients were asked to open their eyelids as wide as possible. The distance between the midpoint of the upper and lower eyelids (accurate to the millimeter) was measured. Pupil diameter was measured using a ruler under stationary indoor lights, and all testing rooms had the same level of light. The physician directly tested the patient’s light reflex with a flashlight.

The procedure involves initially assessing the patient (patients did not use acetylcholinesterase inhibitors, steroids, or any other immunosuppressants) using CDT to determine the affected EOMs and the degree of involvement based on computer-generated images and data. Subsequently, OMNPS is employed to measure the range of eye movement, focusing primarily on recording the primary and secondary gaze positions of the patient. By cross-referencing these two methods, the potential for misdiagnosis is minimized.

In this study, statistical analysis was performed using GraphPad Prism (version 10.1.1) software. The associations between affected EOMs involvement and the total number of involved EOMs, as well as affected EOMs and the Neostigmine test positivity, were assessed using chi-squared tests. Unilateral or bilateral EOM involvement was examined by the Mann–Whitney test. Pearson regression analysis explored the relationship between affected EOMs and Neostigmine test positivity. The significance was set at *p* < 0.05.

## 3 Results

The study included 43 patients, with a range in disease duration from 2 days to 10 years (Q25 = 13 days, Q50 = 30 days, and Q75 = 120 days). The age of the patients ranged from a minimum of 38 years to a maximum of 78 years, with a mean age of 55.53 years. A total of 36 patients underwent thymic CT scans, with no positive findings in any of the cases. Among the patients, 30 were male individuals and 13 were female individuals. In total, there were 113 affected EOMs identified. Tables 1, 2 show the details of the affected EOMs.

**Table 1:** Details of EOM involvement.

SO	IO	LR	MR	IR	SR	LPS	Patient	LPS	SR	IR	MR	LR	IO	SO
			+				A1	+			+			
		+					A2							
							A3				+			
		+				+	A4					+		
		+					A5	+						
			+				A6				+			
						+	A7	+						
							A8				+			
							A9			+				
					+		A10			+				
			+			+	A11	+	+		+	+		
		+			+		A12			+				
		+	+	+	+	+	A13	+						
				+			A14	+				+		
							A15	+	+	+	+		+	
		+	+	+	+		A16	+			+	+		
+							A17							
					+	+	A18							
						+	A19	+						
		+					A20					+		
		+					A21							
							A22	+	+	+	+		+	
						+	A23							
						+	A24	+						
						+	A25	+						
			+	+	+		A26	+						
		+					A27				+			
							A28	+						
							A29	+						
			+			+	A30							
						+	A31			+				
							A32	+	+		+			
							A33	+						
						+	A34							
				+			A35							
							A36	+	+	+	+		+	
+							A37							
						+	A38	+	+	+		+		
						+	A39	+						
		+	+		+	+	A40	+	+	+	+	+		
						+	A41	+						
		+				+	A42							
							A43	+	+			+		

**Table 2:** EOM involvement.

	SUM	LPS	SR	IR	MR	LR	IO	SO
R	63	23	8	9	12	8	3	0
L	50	17	7	5	8	11	0	2
SUM	113	40	15	14	20	19	3	2

**Figure 3:**
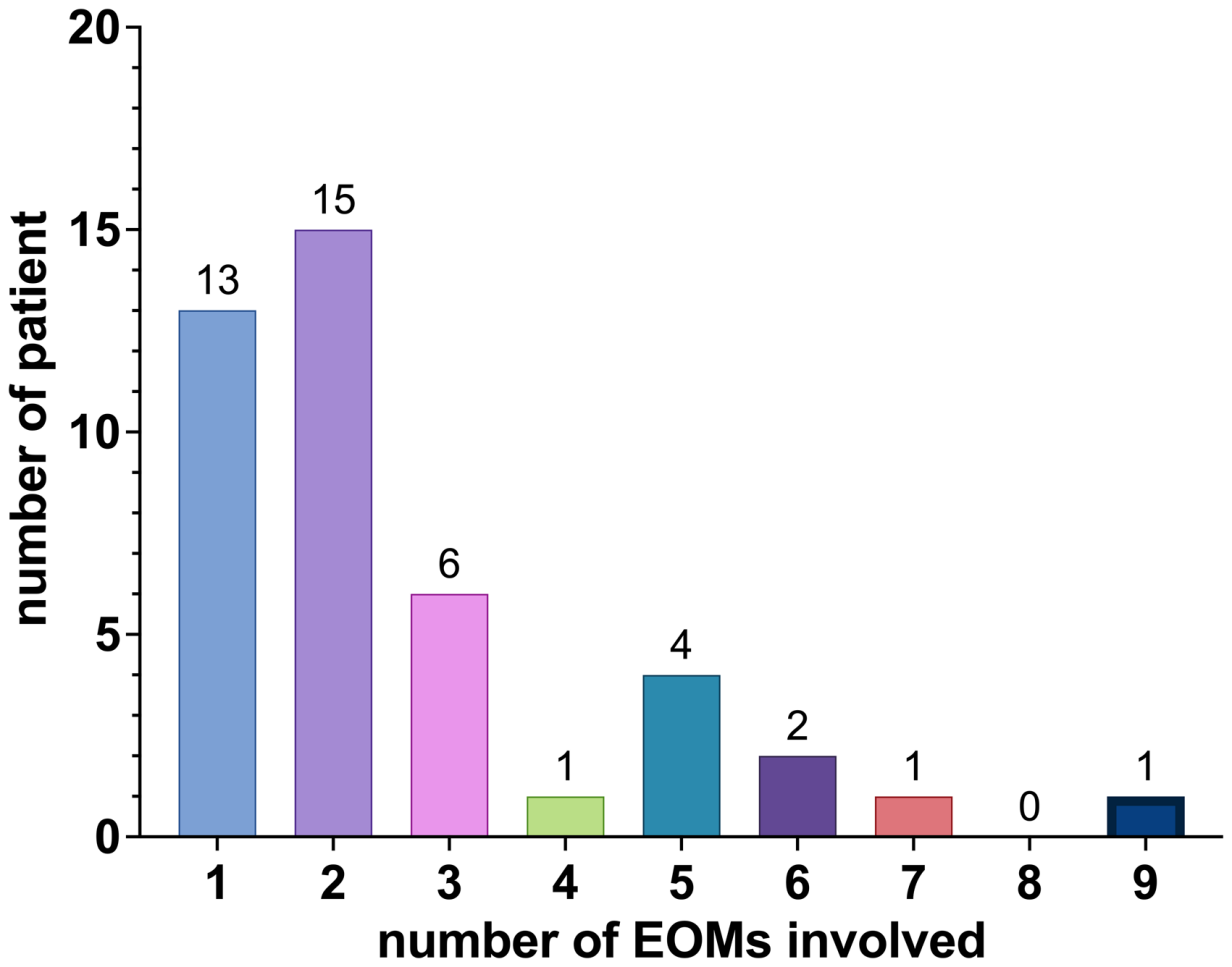
Distribution of EOM involvement.

The distribution of EOMs involvement among the patients is summarized as follows: 13 patients had involvement in 1 EOM, 15 patients had involvement in 2 EOMs, 6 patients had involvement in 3 EOMs, 1 patient had involvement in 4 EOMs, 4 patients had involvement in 5 EOMs, 2 patients had involvement in 6 EOMs, 1 patient had involvement in 7 EOMs, and 1 patient had involvement in 9 EOMs, as shown in Figure 3.

**Figure 4:**
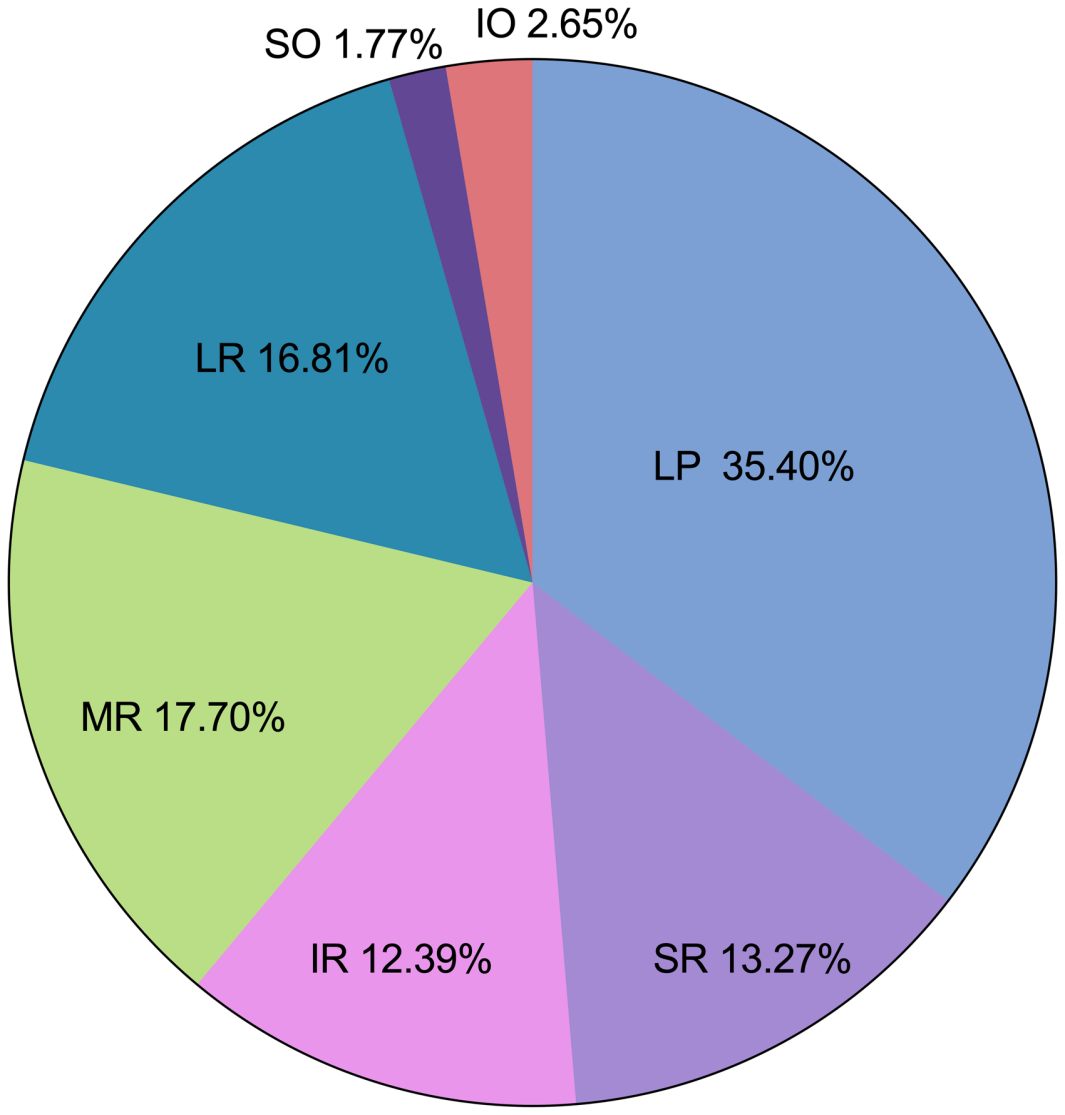
Different EOM involvement.

Among the 113 affected EOMs, the specific distribution was as follows: levator palpebrae superioris (LPS) (40 occurrences, 35.40%), superior rectus (SR) (15 occurrences, 13.27%), inferior rectus (IR) (14 occurrences, 12.39%), MR (20 occurrences, 17.70%), LR (19 occurrences, 16.81%), superior oblique (SO) (2 occurrences, 1.77%), and IO (3 occurrences, 2.65%), as shown in Figure 4; The observed distribution of EOM involvement differs significantly from the expected distribution of average EOM involvement (*p* < 0.0001).

**Figure 5:**
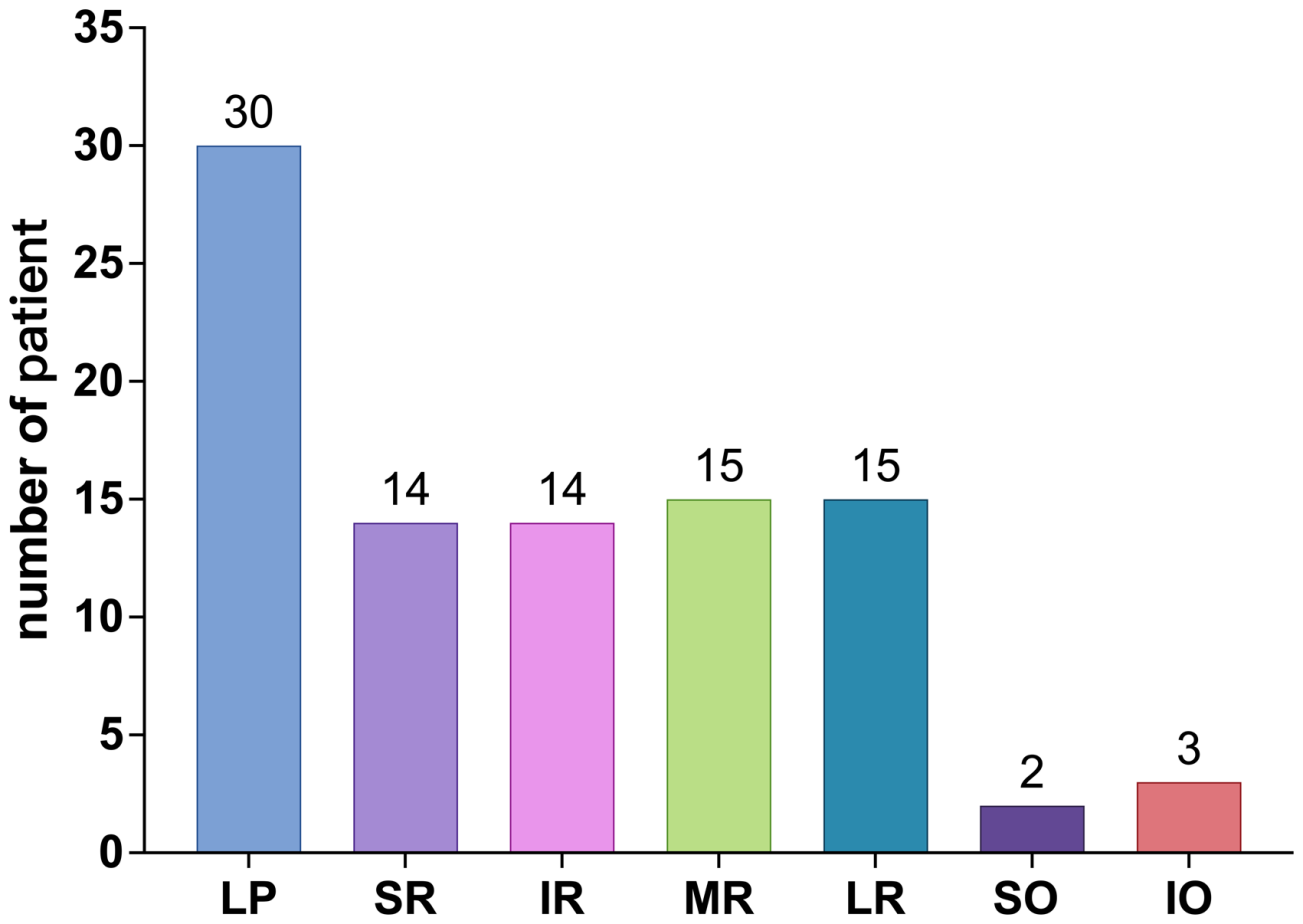
Distribution of different EOM involvement.

Further breakdown of EOM involvement revealed the following occurrences: LPS (30 cases), SR (14 cases), IR (14 cases), MR (15 cases), LR (15 cases), SO (2 cases), and IO (3 cases), as shown in Figure 5. The observed distribution of cases differs significantly from the expected distribution of average EOM involvement (*p* < 0.05).

From Figures 4, 5, it can be observed that the LPS exhibits the highest incidence rate, followed by the four rectus muscles, with their incidence rates being nearly equal, while the two oblique muscles have a very low incidence rate.

**Figure 6:**
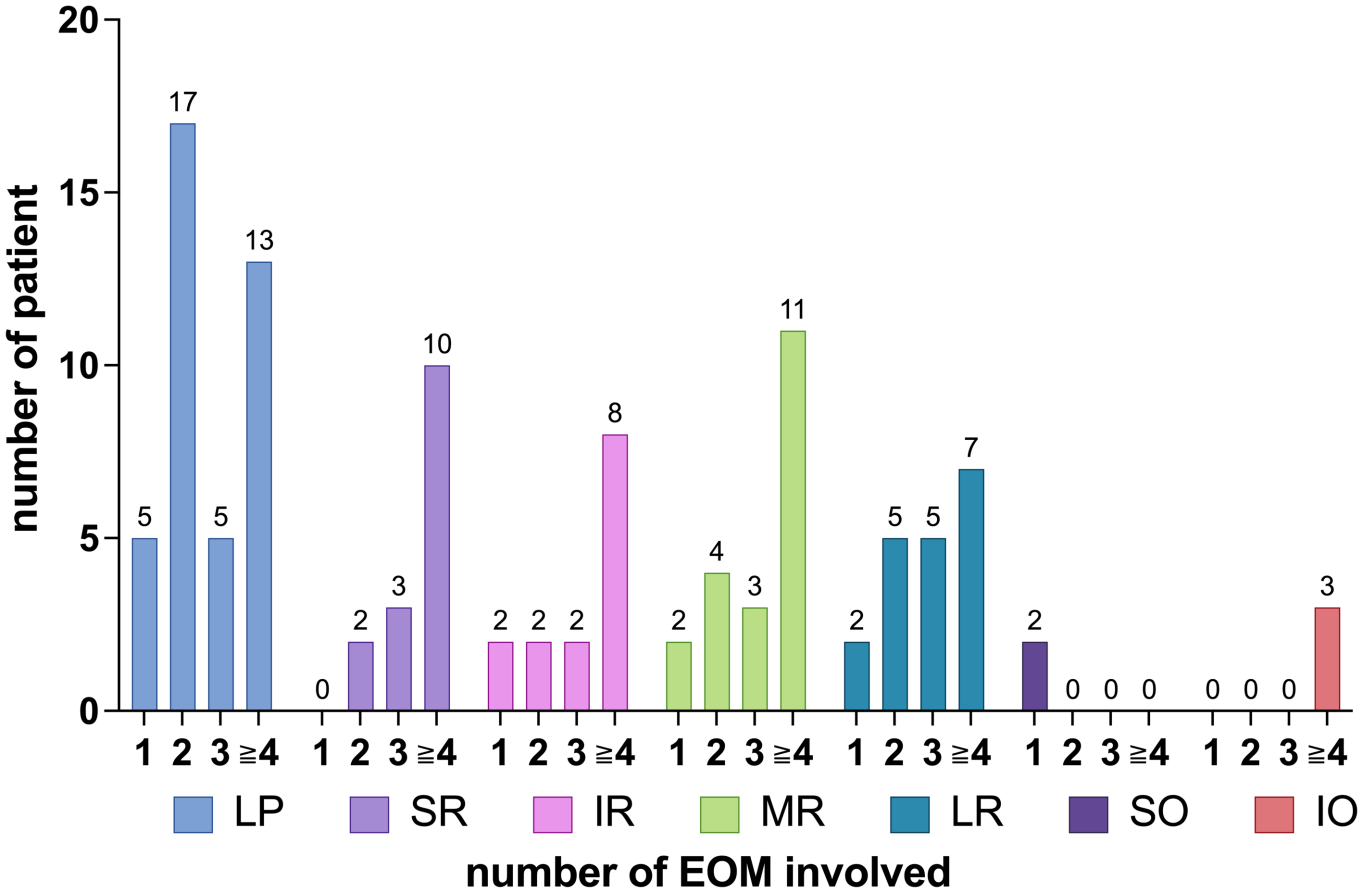
Distribution of EOM involvement varied with an increasing number of affected EOMs.

The distribution pattern of EOM involvement varied with an increasing number of affected EOMs, as shown in Figure 6. Figure 6 shows that, for SR involvement, when comparing the involvement with one muscle to that of more than one muscle, the *p*-value is 0.133 > 0.05. For SO involvement, when comparing the involvement with one muscle to that of more than one muscle, the *p*-value is <0.0001. Regarding IO involvement, when comparing the involvement with more than three muscles to that of three or fewer muscles, the *p*-value is 0.0573 > 0.05.

The positivity rates of the Neostigmine test were 89.19% (33 out of 37), ACHR antibody detection was 59.38% (19 out of 32), and repetitive nerve stimulation was 34.38% (11 out of 32).

**Figure 7:**
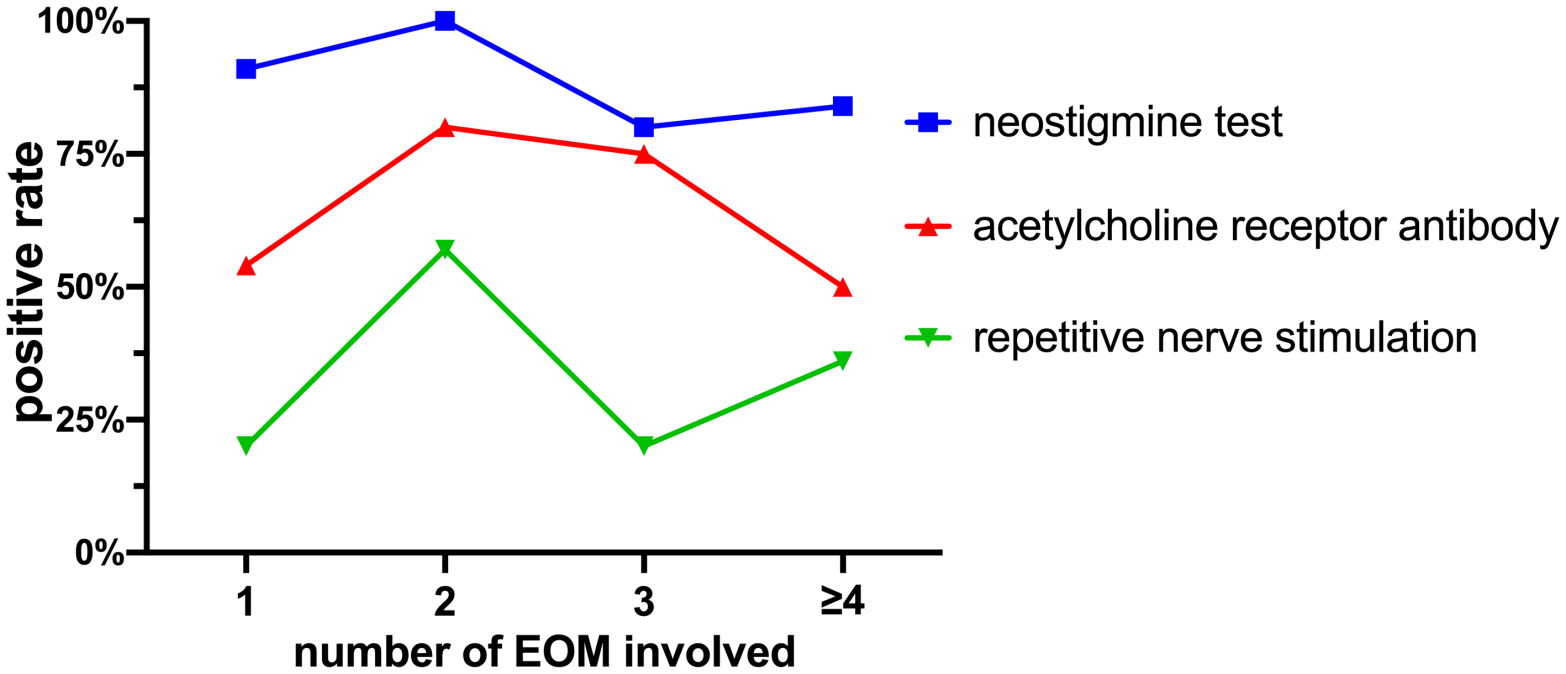
Positivity rates of the Neostigmine test, ACHR antibody detection, and repetitive nerve stimulation.

The relationship between the number of affected muscles and the positivity rates of these tests is shown in Figure 7. Pearson regression analysis revealed a correlation between the number of affected extraocular muscles and the positivity rate of the Neostigmine test (*r* = 0.6544, *p* = 0.3456 > 0.05).

## 4 Discussion

### 4.1 The pattern of EOM involvement

Previous studies have only identified a higher incidence rate in a specific EOM or a certain category of EOM, without finding a unique EOM involvement pattern specific to OMG. Based on our data analysis, we have identified the following patterns: ① The involvement of the EOMs is not uniform. The LPS muscle is most commonly affected, followed by the four rectus muscles, with the oblique muscles being less frequently involved; ② The IO muscle commonly appears in combinations involving four or more affected EOMs. This trend gives us a hint that a person who shows the symptoms described above should be considered a possibility of OMG.

Our observations differ significantly from those of other studies, primarily due to variations in the tools used for detection. Marie Cleary (13) employed the Stand diagrammatic method published in 1993, while Tang (15) utilized the Maddox rod and cover test. These methods are outdated and comparatively less accurate, leading to discrepancies in the findings.

### 4.2 LPS

The higher susceptibility of the LPS compared with other EOMs is indicated by both the number of affected patients and the number of affected muscles. Presently, when evaluating ptosis of the LPS, fatigue tests (repetitive eye opening and closing or sustained upward gaze for 30 s) or ice pack tests (19) are frequently considered more informative as preliminary examinations for OMG. However, in clinical practice, the interpretation of fatigue tests or ice pack tests for evaluating EOM involvement may not be as straightforward as directly observing LPS ptosis, potentially resulting in missed diagnoses. Moreover, some research studies (20) indicate that ptosis and diplopia are the predominant initial symptoms, with a minority of patients remaining in the OMG stage permanently, while approximately 90% of patients undergo disease progression within 2 years of symptom onset. However, we found that ptosis is more predominant than diplopia. In some OMG cases, diplopia symptoms may progressively be replaced by visual suppression (21), leading patients to mistakenly assume that their diplopia has resolved, resulting in missed opportunities for early diagnosis and intervention in MG. In cases where EOM involvement is mild, particularly affecting the MR or LR, the temporal visual field is primarily impacted, and patients may not report diplopia if the affected visual field falls within the suppressed area (22). Moreover, the LPS is anatomically positioned closer to the surface compared with other EOMs, and it occupies a distinct microenvironment relative to the others. In addition, there needs more further study on why.

### 4.3 Study on the tendency of unilateral/bilateral onset

By dividing the total occurrences of a particular EOM by the number of patients affected in that muscle, a value ranging from ≥1 to ≤2 is obtained. The value closer to 1 indicates a higher tendency for unilateral onset, while closer to 2 suggests a higher tendency for bilateral onset. In this study, the values for EOM are shown in Table 3. Table 3 demonstrates the tendency values of LPS and MR as 1.33, indicating a higher probability of bilateral involvement compared with other EOMs (*p* < 0.05). Conversely, IR, SO, and IO have values of 1, SR has a value of 1.07, and LR has a value of 1.27, indicating a higher likelihood of unilateral onset (*p* < 0.05) compared with other EOMs. These results conflict with the findings by Marie Cleary’s (13) regarding bilateral SR involvement in OMG patterns. The discrepancy in these unilateral and bilateral tendencies remains unexplained, suggesting a potential difference in the microenvironment, particularly immunologically, of similar EOMs between the left and right eyes. Further molecular biology research is required to delve into the underlying reasons behind these results.

**Table 3:** Occurrence tendency of EOMs unilaterally and bilaterally.

EOM	LPS	SR	IR	MR	LR	SO	IO
Occurrences	40	15	14	20	19	2	3
Cases	30	14	14	15	15	2	3
Rate	1.33	1.07	1	1.33	1.27	1	1

### 4.4 Neostigmine test positivity rate

Studies have demonstrated that the Neostigmine test exhibits a sensitivity of 93.4% in detecting OMG (23). In our study, we found a similar Neostigmine test positivity rate of 89.19%. Figure 7 illustrates that as the cumulative count increases, the positivity rate of the Neostigmine test experiences fluctuations while displaying an overall declining trend. Through Pearson regression analysis, a moderate positive linear correlation (*r* = 0.6544, *p* = 0.3456 > 0.05) was observed between the number of affected extraocular muscles and the positivity rate of the Neostigmine test. However, this correlation did not reach statistical significance, possibly due to the lack of detailed classification for cases involving more than four affected muscles in our study. However, the underlying mechanism involves the destruction of acetylcholine receptors on the postsynaptic membrane due to the presence of acetylcholine receptor antibodies (21). Over time, the quantity of these receptors gradually diminishes. Once it reaches a certain threshold, the binding of acetylcholine to the synaptic cleft becomes insufficient to generate the threshold potential required for muscle contraction, leading to impaired muscle contraction (15). In certain cases, diagnostic methods that rely on modulating the quantity of acetylcholine in the synaptic cleft, such as fatigue tests, ice pack tests, and the Neostigmine test, may yield negative results. Consequently, it is plausible to speculate that the positivity rate of the Neostigmine test might indicate the severity of MG to some extent, which means that OMG patients with negative Neostigmine test results could potentially signify a more severe condition.

### 4.5 Different positivity rates of the neostigmine test in subgroups

Wang et al. (24), examining the progression of OMG to GMG, discovered that the presence of isolated ptosis or diplopia indicates an earlier onset of progression compared with the combination of ptosis and diplopia. Sivesh K Kamarajah (25) pointed out that seropositive AChR autoantibody status and early age of disease onset were independent predictive risk factors of generalization. Based on these findings, patients can be categorized into three subgroups according to the involvement of the LPS: a subgroup with isolated diplopia (only diplopia), a subgroup with isolated LPS involvement (only ptosis), and a subgroup with simultaneous involvement of ptosis and diplopia. In those groups, AChR antibody positive rates were studied. Tables 4, 5 show the AChR antibody positive rates of those subgroups. Therefore, it cannot be conclusively stated that diplopia and ptosis exhibit a significant disparity in Neostigmine test positivity rates. These symptoms do not serve as definitive markers of disease severity. Additionally, the correlation between diplopia and/or ptosis and the progression of OMG will be explored in a separate article.

**Table 4:** AChR antibody positive rates of subgroups.

Subgroup	Only diplopia /no ptosis	Diplopia and ptosis	Only ptosis /no diplopia
Patient	12	15	10
Positive rate	100%	86.67%	80.00%

**Table 5:** AChR antibody positive rates of subgroups.

	Diplopia	No diplopia	*p*-value	OR value	95%CI
Positive	25	8	0.2917 > 0.05	3.125 > 1	(0.377, 25.920)
Negative	2	2			
	Ptosis	No ptosis			
Positive	21	12	0.2823 > 0.05	0.1911 < 1	(0.009, 3.853)
Negative	4	0			

This study has certain limitations, including its retrospective design, limited sample size, and potential data lag. Moreover, most patients underwent only diagnostic procedures that are sufficient for confirmation and did not undergo all available tests and examinations, and this further reduced the sample size for the analysis of test results, potentially introducing bias into the results. During our follow-up process, a small subset of patients experienced exacerbation or progression; however, we did not incorporate their subsequent changes into the study.

## 5 Conclusion

The involvement of the EOMs is not uniform. The LPS exhibits the highest incidence rate, followed by the four rectus muscles, with their incidence rates being nearly equal, while the two oblique muscles have a very low incidence rate.

The IO muscle commonly appears in combinations involving four or more affected EOMs.

LPS and MR show a higher inclination toward bilateral involvement.

### 5.1 Outlook

In the next step, we will expand our sample size to validate the conclusion of this study. Additionally, we will include the analysis of the degree of eyelid ptosis, the severity of diplopia, and the time points of progression from OMG to GMG in our research.

## Data availability statement

The original contributions presented in the study are included in the article/supplementary material, further inquiries can be directed to the corresponding author.

## Ethics statement

The studies involving humans were approved by the Ethics Committee of the First Affiliated Hospital of Harbin Medical University of China (approval number: 201452). The studies were conducted in accordance with the local legislation and institutional requirements. The participants provided their written informed consent to participate in this study.

## Author contributions

Y-fF: Methodology, Software, Visualization, Writing – original draft, Writing – review & editing. S-jT: Writing – review & editing. YL: Software, Writing – review & editing. X-mL: Writing – review & editing. T-jL: Software, Writing – review & editing. L-yZ: Writing – review & editing.
